# Energy transmission in mechanically ventilated children: a translational study

**DOI:** 10.1186/s13054-020-03313-7

**Published:** 2020-10-07

**Authors:** Martin C. J. Kneyber, Stavroula Ilia, Alette A. Koopman, Patrick van Schelven, Jefta van Dijk, Johannes G. M. Burgerhof, Dick G. Markhorst, Robert G. T. Blokpoel

**Affiliations:** 1 Division of Paediatric Critical Care Medicine, Department of Paediatrics, Beatrix Children’s Hospital, University Medical Center Groningen, The University of Groningen, Internal Postal Code CA 80, P.O. Box 30.001, 9700 RB Groningen, the Netherlands; 2 Critical Care, Anesthesia, Peri-operative Medicine & Emergency Medicine (CAPE), The University of Groningen, Groningen, the Netherlands; 3 Pediatric Intensive Care Unit, University Hospital Heraklion, University of Crete, Crete, Greece; 4 Department of Epidemiology, University Medical Center Groningen, The University of Groningen, Groningen, the Netherlands; 5 Pediatric Intensive Care Unit, Emma Children’s Hospital, Amsterdam UMC, Amsterdam, the Netherlands

**Keywords:** Mechanical power, Energy per breath, Mechanical ventilation, Pediatric, Ventilator-induced lung injury (VILI)

## Abstract

**Background:**

Recurrent delivery of tidal mechanical energy (ME) inflicts ventilator-induced lung injury (VILI) when stress and strain exceed the limits of tissue tolerance. Mechanical power (MP) is the mathematical description of the ME delivered to the respiratory system over time. It is unknown how ME relates to underlying lung pathology and outcome in mechanically ventilated children. We therefore tested the hypothesis that ME per breath with tidal volume (Vt) normalized to bodyweight correlates with underlying lung pathology and to study the effect of resistance on the ME dissipated to the lung.

**Methods:**

We analyzed routinely collected demographic, physiological, and laboratory data from deeply sedated and/or paralyzed children < 18 years with and without lung injury. Patients were stratified into respiratory system mechanic subgroups according to the Pediatric Mechanical Ventilation Consensus Conference (PEMVECC) definition. The association between MP, ME, lung pathology, and duration of mechanical ventilation as a primary outcome measure was analyzed adjusting for confounding variables and effect modifiers. The effect of endotracheal tube diameter (ETT) and airway resistance on energy dissipation to the lung was analyzed in a bench model with different lung compliance settings.

**Results:**

Data of 312 patients with a median age of 7.8 (1.7–44.2) months was analyzed. Age (*p* <  0.001), RR *p* <  0.001), and Vt <  0.001) were independently associated with MPrs. ME but not MP correlated significantly (*p* <  0.001) better with lung pathology. Competing risk regression analysis adjusting for PRISM III 24 h score and PEMVECC stratification showed that ME on day 1 or day 2 of MV but not MP was independently associated with the duration of mechanical ventilation. About 33% of all energy generated by the ventilator was transferred to the lung and highly dependent on lung compliance and airway resistance but not on endotracheal tube size (ETT) during pressure control (PC) ventilation.

**Conclusions:**

ME better related to underlying lung pathology and patient outcome than MP. The delivery of generated energy to the lung was not dependent on ETT size during PC ventilation. Further studies are needed to identify injurious MErs thresholds in ventilated children.

## Background

Mechanical ventilation (MV) as life-saving intervention is ubiquitous in pediatric intensive care units (PICU) but may simultaneously lead to ventilator-induced lung injury (VILI) [1]. Experimental and clinical studies have identified factors contributing to VILI to include volutrauma (i.e., delivery of large tidal volumes (Vt) as a surrogate for lung strain), lung stress, and atelectrauma (i.e., the repetitive opening and closure of alveoli) albeit that their exact role in the pediatric setting is not well understood [1, 2].

Mechanical power (MPrs) has been proposed as a measure and potential driver of VILI [3]. MPrs is the energy per breathing cycle multiplied by ventilation frequency [4]. It can be calculated during volume-controlled (VC) ventilation using the respiratory rate (RR), peak inspiratory pressure (PIP), plateau pressure (Pplat), and positive end-expiratory pressure (PEEP) [5]. Although MPrs has been associated with mortality in ARDS patients, it is not well-established if MPrs should be normalized to lung volume or predicted bodyweight and how much power is delivered to the lung [3, 6–8].

It has not been studied how the conceptual framework of power translates to mechanically ventilated children. Obviously, two key components of MPrs (i.e., Vt and RR) are age-dependent (i.e., the older the child, the larger the Vt and the lower the RR), making those injurious thresholds for MPrs not uniform across the entire pediatric spectrum. Furthermore, pediatric critical care practitioners predominantly use a pressure-controlled (PC) mode of ventilation whereas the concept MPrs is based on VC ventilation although Becher et al. have proposed an approach to measure MPrs during PC ventilation using peak inspiratory pressure (PIP) instead of Pplat [9–11]. It needs to be studied if these modifications can be used to study mechanical power in children. Aside from these methodological aspects, infants and young children are generally ventilated with small endotracheal tube (ETT) sizes with high gas flow and high ventilation rate, all contributing to resistance (Rrs) [12]. Added to that, especially young children often suffer from disease conditions characterized by increased airway resistance such as viral bronchiolitis or pneumonia [13]. It is thus unclear how much of the power is delivered to the lungs and how much is dissipated in the native airways.

We therefore sought to explore how MPrs and energy with Vt normalized to bodyweight (thereby eliminating age dependency) correlated with underlying lung pathology and patient outcome. In addition, we wanted to study if ETT size impacted energy delivery to the lung.

## Methods

We analyzed routinely collected demographic, physiological, and laboratory data from deeply sedated and/or paralyzed children < 18 years with and without lung injury on weekdays at 8 am during the first 3 days of MV. Data from patients with obstructive airway disease, documented chronic lung disease, neuromuscular disorders, premature birth with age corrected for post-conceptional age less than 40 weeks, severe traumatic brain injury (i.e., Glasgow Coma Scale < 8), chronic lung disease (i.e., tracheostomy ventilation), and severe pulmonary hypertension, managed with high-frequency oscillation ventilation or with an ETT leakage > 18% were excluded. The severity of the disease was assessed by the 24-h Pediatric Risk of Mortality II (PRISM II) [14]. Patients were stratified according to underlying respiratory system mechanics subgroups proposed by the Pediatric Mechanical Ventilation Consensus Conference (PEMVECC) definition and pediatric acute lung injury consensus conference (PALICC) definition for PARDS [13, 15]. The Institutional Review Board approved the study and waived the need for informed consent. A bench study was performed to study how the ETT affected energy transmission to the lung (see Additional file 1).

Patients were ventilated in a PC ventilation mode, limiting inspiratory pressures < 28–32 cmH_2_O and expiratory Vt (Vt-exp) 5–7 mL/kg actual bodyweight (as there was no obesity in the patient cohort). Vt-exp was measured near the Y-piece in children < 10 kg (VarFlex™, Vyaire, Mettawa, Ill, USA). Mandatory breath rate setting was guided by the underlying pathology and age; the flow-time scalar is carefully monitored to identify appropriate inspiratory time setting and to avoid the development of intrinsic PEEP. The I to E ratio is not fixed. Initial PEEP was 4–6 cm H_2_O and further titrated at the discretion of the attending physician, targeting SpO_2_ 88–92% for patients with lung injury. Unless dictated otherwise, the target pH was > 7.20.

Demographic, physiological, and laboratory data were manually extracted from the patient’s medical record. Ventilator settings and parameters were read from the ventilator. Plateau pressure (Pplat) and quasi-static compliance (Crs) were measured at end-inspiration by a manual inspiratory hold maneuver of 3 s. Metrics for oxygenation included the oxygenation index [OI] ([mean airway pressure × FiO_2_ × 100]/PaO_2_) and if the SpO_2_ was < 98% the oxygen saturation index [OSI] [mean airway pressure × FiO_2_ × 100]/SpO_2_). We calculated ventilator-free days (VFD) through day 28, defined as the number of days within 28 days that a subject is alive and free of MV [16]. Patients were assigned 0 VFD if they remained intubated or died prior to day 28 without remaining extubated for more than 24 h. MPrs was calculated as previously described [5]. The mechanical energy of the respiratory (MErs) was calculated by 0.098 × (Vt × kg^− 1^) × (PIP − [(Pplat − PEEP)/2]).

### Statistical analysis

The normality of data was assessed using the Kolmogorov-Smirnov test. Continuous data are presented as median and 25–75 interquartile range (IQR) and analyzed using the Mann-Whitney *U* test (for comparing two groups) or Kruskal-Wallis test; Spearman correlation coefficient (*r*_*s*_) was calculated to analyze correlations. The *χ*^2^ test with Yates continuity correction was used to analyze categorical data. The primary outcome measure was the duration of mechanical ventilation (MV). Competing risk regression analysis (Fine and Gray model) was used to identify independent contributors to the duration of MV with death as a competing risk [17]. All statistical analyses were performed using software IBM SPSS, v24.0 (IBM Corp., Chicago, III, USA) with *P* <  0.05 considered statistically significant.

## Results

### Correlation MPrs and MErs with underlying lung pathology and patient outcome

Data of 312 patients with a median age of 7.8 (1.7–44.2) months was analyzed (Table 1). Of these, 186 patients (61.5%) were younger than 12 months of age. Primary admission diagnosis was respiratory failure in 102 (32.7%) and cardiac (including post-cardiac surgery) in 81 (26.0%) patients. Sixty-nine (22.1%) patients met PEMVECC criteria for restrictive and 78 (25.0%) for mixed lung disease, and 7.7% of patients met PALICC criteria for PARDS. The duration of MV was 93 (44–163) hours. Thirteen (4.2%) patients died. Patients were ventilated for a median of 17.3 h (11.6–21.2) before the first measurement on day 1 was made.

**Table 1:** Characteristics of the study population of *N* = 312 mechanically ventilated children. Data are presented as median (25–75 interquartile range) or percentage (%) of total. Ventilator-free days (VFD) through day 28 were defined as the number of days within 28 days that a patient was alive and free of mechanical ventilation [16]. Patients were assigned 0 VFD if they remained intubated or died prior to day 28 without remaining extubated for more than 24 h. *Denotes *p* < 0.005 (Kruskal Wallis test). PRISM Pediatric Risk of Mortality; OI oxygenation index

	Patient stratification by Pediatric Mechanical Ventilation Consensus Conference category			
	Normal respiratory system mechanics	Restrictive respiratory system mechanics (i.e., reduced compliance)	Mixed respiratory system mechanics (i.e., reduced compliance and increased resistance)	Cardiac (combination of normal and altered respiratory system mechanics)
	*N* = 84	*N* = 69	*N* = 78	*N* = 81
Baseline patient characteristics				
Age (months) *	49.3 (9.4–122.0)	23.1 (8.0–46.0)	2.0 (1.2–8.5)	2.2 (0.5–6.7)
≤ 12 months (%)	29.78	39.1	84.6	84.0
13–60 months (%)	20.2	34.8	11.5	6.2
> 60 months (%)	50.0	26.1	3.8	9.9
Male gender (%)	61.9	58.0	62.8	63.0
Weight (kg) *	16.5 (8.0–29.5)	12.4 (8.5–17.5)	5.0 (4.0–6.7)	4.2 (3.6–6.9)
PRISM III 24 h score	8.5 (5.0–14.0)	10.0 (6.0–15.0)	11.0 (8.0–15.0)	12.0 (7.0–15.5)
PARDS (%)	0.0	5.8	24.4	1.2
Cstat (cmH_2_O/L/kg) *	0.59 (0.43–0.78)	0.55 (0.39–0.73)	0.34 (0.28–0.44)	0.5 (0.32–0.6)
Baseline ventilator settings				
PIP (cmH_2_O) *	18 (16–22)	20 (17–23)	27 (24–29)	20 (18–23)
Pplat (cmH_2_O) *	16 (14–19)	17 (15–21)	24 (21–26)	18 (16–21)
PEEP (cmH_2_O) *	5 (5–6)	5 (5–6)	6 (5–7)	5 (5–6)
Vt-exp (mL/kg) *	7.6 (6.6–8.6)	7.6 (6.5–8.5)	6.6 (6.1–7.7)	7.5 (6.8–8.3)
Inspiratory time (s) *	0.75 (0.62–0.9)	0.7 (0.6–0.75)	0.55 (0.5–0.6)	0.55 (0.5–0.6)
Mandatory breath rate (/min) *	22 (17–30)	23 (20–30)	40 (32–40)	35 (29–40)
Outcome data				
Ventilation time (h) *	88.3 (42.5–163.4)	69.8 (25.3–179.3)	107.5 (78.0–152.2)	91.5 (32.6–163.1)
VFD day 28	24 (20–26)	24 (19–26)	23 (21–24)	24 (21–26)
PICU mortality (%)	6.0	4.3	2.6	3.7

Data on MPrs was available on the first day of MV for all patients, on day 2 for 169 (53.9%), and on day 3 for 96 (30.0%) patients. We found a direct relationship between age and MPrs (*r*_*s*_ 0.814, *p* <  0.001). Vt-exp (*r*_*s*_ 0.112, *p* <  0.01), weight (*r*_*s*_ 0.837, *p* <  0.001), and RR (*r*_*s*_ − 0.459, *p* <  0.001) were also significantly correlated with MPrs. There was also a significant correlation between MPrs and oxygen saturation index (OSI) (*r*_*s*_ 0.189, *p* <  0.001) but not with oxygenation index (OI) or PaO_2_/FiO_2_ ratio. PEEP was significantly correlated with MPrs (*r*_*s*_ 0.311, *p* <  0.001).

**Fig. 1:**
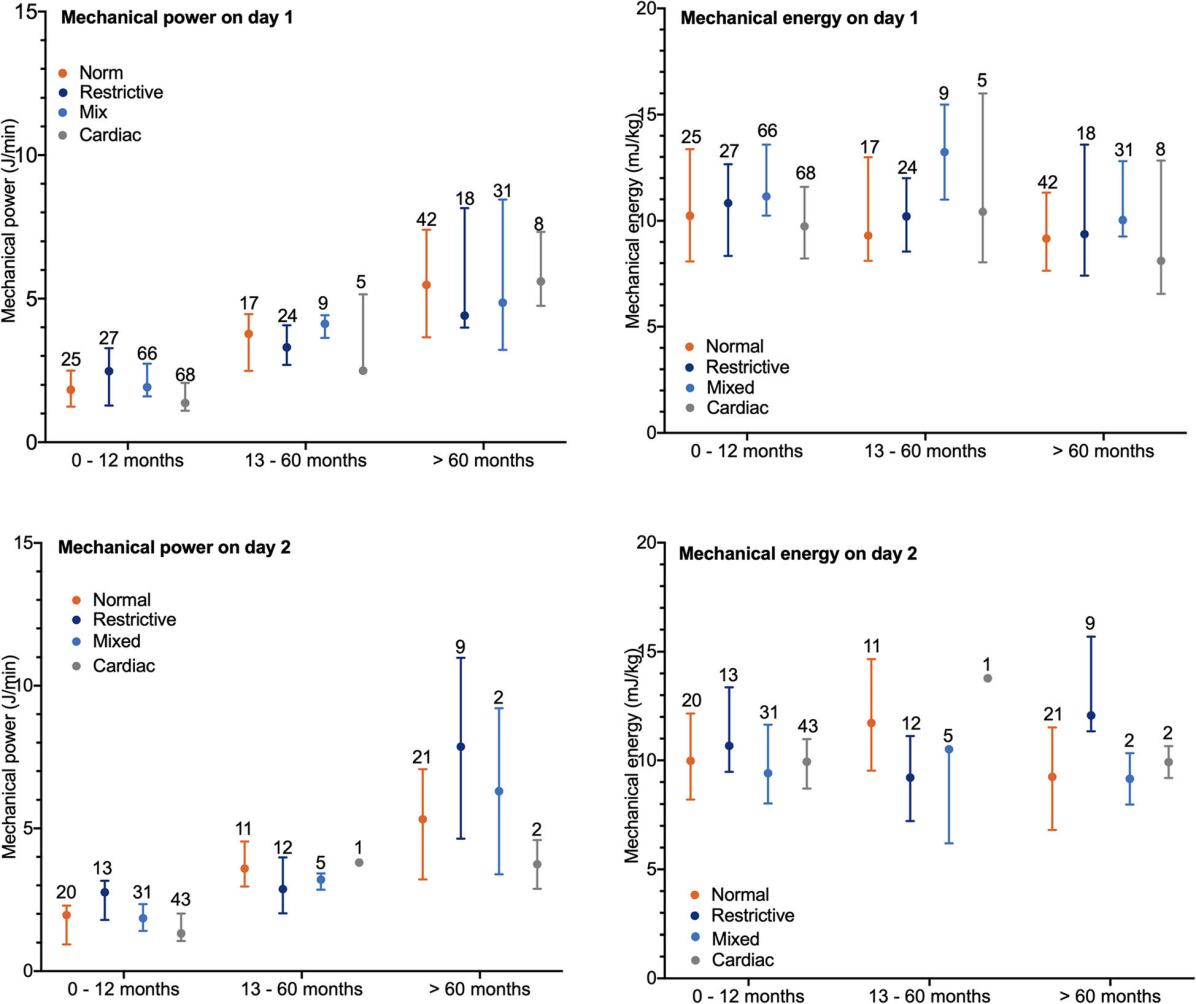
Distribution of mechanical power of the respiratory system (MPrs) on day 1 (upper left panel) and day 2 (lower left panel) and the mechanical energy per breath [MErs] on day 1 (upper right panel) and day 2 (lower right panel) stratified by Pediatric Mechanical Ventilation Consensus Conference (PEMVECC) defined type of respiratory system mechanics. Data are depicted as median (25–75 interquartile range). Absolute values above lines represent number of patients per PEMVECC type

The distribution of MPrs on day 1 was significantly different (*p* <  0.001) across the cohort after stratification by PEMVECC criteria (Fig. 1). Linear regression analysis showed that age in months (*β* − 0.012 [95%CI − 0.018 − − 0.007, *p* <  0.001), RR (*β* 0.047 [95%CI 0.034–0.060, *p* <  0.001), and total Vt (i.e., not normalized to bodyweight) (*β* 10.028 [95%CI 0.024–0.031, *p* <  0.001) were independently associated with MPrs.

Next, we analyzed the mechanical energy per breath (MErs) [expressed as mJ/kg] after normalizing Vt to actual bodyweight. For the whole cohort, MErs was 10.2 (8.2–12.2) mJ/kg. MErs was not significantly correlated with age in months. There was a significant correlation between MErs and OI (*rs* 0.412, *p* <  0.001), OSI (*rs* 0.438, *p* <  0.001), and PaO_2_/FiO_2_ ratio (*rs* − 0.263, *p* <  0.001). The distribution of MErs was significantly different (*p* < 0.001) across the cohort after stratification by PEMVECC criteria (Fig. 1b). MErs was 9.7 mJ/kg (8.0–12.3) in patients with normal lung mechanics, 10.7 mJ/kg (8.6–12.5) in patients with the restrictive disease, and 10.6 mJ/kg (8.6–12.5) in patients with mixed disease although this difference did not reach statistical significance. MErs but not MPrs was significantly higher in non-survivors (11.7 [8.9–15.4] vs 10.1 mJ/kg [8.2–12.1], *p* = 0.016).

Patients with new PARDS during the first 3 days of MV had significantly higher MPrs (3.2 [1.7–8.3] vs 2.4 [1.5–3.7] J/L, *p* = 0.003) and MErs (11.1 mJ/kg [9.6–14.0] vs 10.0 [8.2–12.0], *p* = 0.001) than those without PARDS. Analysis of patients with PARDS showed that MPrs or MErs was not significantly correlated with VFD-28 or different between survivor and non-survivors.

Univariate analysis showed that for the entire cohort only MErs was significantly correlated with VFD (*r*_*s*_ − 0.243, *p* < 0.001). After stratification by age, we found a significant correlation between MPrs, MErs, and VFD in two age categories (0–12 months and 1–5 years) but not in children > 5 years. Competing risk regression analysis adjusting for PRISM III 24 h score, age (in months), and PEMVECC stratification showed that MPrs on day 1 or day 2 of MV was not independently associated with VFD, whereas MErs on day 1 or day 2 was (*p* < 0.001).

### Bench model testing

The median mechanical energy measured in the lung was significantly higher with VC (4.8 [2.7–8.2] than with PC ventilation (2.3 [1.2–3.4] mJ/kg, *p* < 0.001). We also found that the mechanical energy lost due to resistive properties of the respiratory system was significantly higher with VC (7.4 [4.7–12.4] than with PC (3.8 [1.6–7.2] mJ/kg, *p* < 0.001). ETT size was significantly correlated with mechanical energy in the lung (*r*_*s*_ − 0.055, *p* < 0.01) and mechanical energy lost due to resistive properties of the respiratory system (*r*_*s*_ − 0.050, *p* < 0.01). Multivariate analysis showed that for VC but not PC, ETT size (*p* < 0.001) was independently associated with the percentage of MEventilator delivered to the lung, irrespective of compliance or resistance settings (Table 2).

**Table 2:** Results from multivariate linear regression analysis from bench model testing data examining the independent contribution of categorized endotracheal tube (ETT) size (0 [ETT 3 mm] to 5 [ETT 8 mm]), categorized compliance (1 [normal] to 5 [severely low]), categorized resistance (1 [no resistance] to 3 [high]), categorized tidal volume (in mL/kg) for VC ventilation or pressure above PEEP for PC ventilation, and categorized inspiratory time (1 [− 20% compared to age-appropriate] to 3 [+ 20% compared to age-appropriate]) to the percentage of mechanical energy generated by the ventilator (calculated by [(0.098 × (Vt × kg^− 1^) × (PIP – [(Pplat – PEEP)/2])] with ventilator parameters measured at the Y – piece) that is delivered to the lungs (measured by integrating the dynamic pressure – volume curve in the test lung)

	*β*	95% CI	*P* value
A: Percentage of energy dissipated to the lung during VC ventilation			
ETT size (mm)	− 1.391	− 1.908; − 0.0874	< 0.001
Vt (mL/kg)	3.294	2.592;3.997	< 0.001
Compliance (mL/cmH_2_O/kg)	13.326	12.931;13.720	< 0.001
Resistance	− 8.061	− 8.722; − 7.401	< 0.001
Inspiratory time (s)	2.754	2.078;3.430	< 0.001
Inspiratory flow (L/min)	− 0.041	− 0.092;0.009	0.110
B: Percentage of energy dissipated to the lung during PC ventilation			
ETT size (mm)	0.090	− 0.299;0.478	0.650
Pressure above PEEP (cmH_2_O)	0.602	0.444;0.761	< 0.001
Compliance (mL/cmH_2_O/kg)	11.130	10.662;11.598	< 0.001
Resistance	−10.206	− 11.000; − 9.413	< 0.001
Inspiratory time (s)	4.322	3.528–5.115	< 0.001

## Discussion

This is the first pediatric study investigating energy transmission during mechanical ventilation. We found that mechanical energy per breath correlated with underlying lung pathology and patient outcome. Bench testing showed that during PC ventilation, ETT size itself did not affect energy delivery to the lung. Our data provide support to further exploring the conceptual framework of energy transmission during MV in the pediatric setting.

Recurrent delivery of tidal energy inflicts VILI when stress and strain exceed the limits of tissue tolerance. Experimental work linked mechanical power > 12 J/min to the development of VILI [18–21] and adverse outcome in clinical studies in adults [7, 8, 22]. To date, there are no pediatric studies that have examined such relationship. Although it is not clear which component of mechanical power contributes the strongest to lung damage, none of these components have been extensively studied in mechanically ventilated children and the relationship between mechanical ventilation and VILI in children remains unclear [2]. In fact, high Vt has been linked with better outcome in observational studies of children with acute lung injury, making it impossible to identify a specific Vt threshold to be associated with adverse outcomes [23–25]. Driving pressure has not been studied in children so far except for two observational studies reporting an association between a surrogate driving pressure (i.e., pressure gradient calculated by subtracting PEEP from the PIP measured under dynamic flow conditions) [26, 27].

Our study shows indicates that assessing the mechanical energy per breath seems appropriate when studying the effects of energy transmission on patient outcome instead of using the proposed formula for MPrs. Not only did we find values for MPrs far below previously published injurious thresholds in adults, we also found that there was no correlation with underlying pathology based on the PEMVECC criteria [5, 18]. In fact, we found the highest values for MPrs among patients with no lung pathology which may be perceived as counterintuitive. A high tidal strain is a prerequisite for poor tissue damage, underscoring the injurious role of high Vt [18, 20]. We propose that our observations may largely be caused by the age-dependency of Vt and RR. From a developmental perspective, the older the child becomes, the larger the Vt gets and the lower the RR is, indicating that higher values of MPrs are automatically found in older children and thus making comparisons across the entire pediatric spectrum impossible. Furthermore, it could be surmised that this is also due to differences in lung size. When normalized to height instead of bodyweight, younger children have a relatively larger lung surface area than adults [2]. However, the actual delivered Vt in relation to the amount of inflatable lung volume will then be lower when still normalized to bodyweight leading to a lower lung strain compared with adults [28]. Aside from this, PC is the predominant ventilatory mode in pediatrics. Raw is not constant in PC because of the decelerative flow pattern; thus, (Ppeak – Pplat)/Flow cannot function as a proxy for Raw and Vt/Tinsp not for flow [29]. We overcame this applying an inspiratory hold to generate Pplat and found that the energy transmitted to the lung remained lower during PC than during VC ventilation. One obvious explanation would be the significantly lower delivered Vt during PC when setting an inspiratory pressure. Interestingly, multivariate testing showed that ETT size was not independently associated with the percentage of energy that was transmitted to the lung during PC ventilation, further supporting the use of mechanical energy rather than mechanical power in the pediatric setting where PC ventilation is very common. Nonetheless, although mechanical energy per breath showed an observed a better relationship with lung pathology based on the PEMVECC criteria, injurious thresholds obviously need to be identified in future studies.

Our study has several limitations. First, our clinical study was designed a single-center study that may limit the generalizability of our findings, although our unit is comparable to most large units globally. In addition, the clinical relevance of mechanical has only been studied in ARDS, but our cohort included only a small group of PARDS patients. Second, although lung-protective ventilation is guided by a unit-specific clinical algorithm guiding pressure and Vt setting, setting the level of PEEP was not dictated by the ARDS Network grid. Recently, it has been shown that not adhering to the grid was associated with increased mortality, although these findings need to be universally confirmed [30]. Third, although we showed that MErs on day 1 or day 2 was independently associated with total ventilation time after adjusting for age, disease severity, and PEMVECC lung pathology type, this does not mean that there is a causative relationship between MErs and patient outcome since other confounders may not have been picked up. For example, our unit has a low threshold for using HFOV as an alternative mode of ventilation which may impact total ventilation time [31, 32]. Also, there was no use of extubation readiness testing, thereby affecting total ventilation time [33]. Importantly, it remains to be studied if mechanical energy may be interpreted as a driver of the outcome. Fourth, our bench test represented a simplification of the actual clinical situation because by design, a bench only represents homogeneous lung conditions.

## Conclusions

Compared with mechanical power, mechanical energy per breath correlates better with underlying lung pathology and patient outcome in mechanically ventilated children. Delivery of the generated energy was not dependent on endotracheal tube size during pressure-controlled ventilation. Future studies are needed to identify injurious thresholds of mechanical energy.

## Supplementary information

**Additional file 1.** Electronic Supplemental Material To Energy transmission in mechanically ventilated children: a translational study. Methodology of the bench test.

## Data Availability

The datasets used and/or analyzed during the current study are available from the corresponding author on reasonable request.

## Abbreviations

ARDS: Acute respiratory distress syndrome
Crs: Compliance of the respiratory system
DP: Driving pressure
ETT: Endotracheal tube
MElung: Mechanical energy measured in the test lung
MErs: Mechanical energy of the respiratory system
MEventilator: Mechanical energy measured in the ventilator
MPrs: Mechanical power of the respiratory system
MV: Mechanical ventilation
OI: Oxygenation index
OSI: Oxygen saturation index
PALICC: Pediatric Acute Lung Injury Consensus Collaborative
PARDS: Pediatric acute respiratory distress syndrome
PC: Pressure control
PEEP: Positive end-expiratory pressure
PICU: Pediatric intensive care unit
PIP: Peak inspiratory pressure
Pplat: Plateau pressure
PRISM: Pediatric risk of mortality
RR: Respiratory rate
Rrs: Resistance of the respiratory system
Tinsp: Inspiratory time
VC: Volume control
VILI: Ventilator-induced lung injury
Vt: Tidal volume
