# Supplementary material for: Energy transmission in mechanically ventilated children: a translational study
Source: Crit Care. 2020 Oct 7;24:601. doi: 10.1186/s13054-020-03313-7 (PMC7539278; doi:10.1186/s13054-020-03313-7)
Supplement: Supplementary file 1 — Additional file 1. Electronic Supplemental Material To Energy transmission in mechanically ventilated children: a translational study. Methodology of the bench test. [file 13054_2020_3313_MOESM1_ESM.docx]

Electronic supplemental material to

**Energy transmission in mechanically ventilated children: a translational study**

Martin C.J. Kneyber, MD PhD FCCM (1,4), Stavroula Ilia, MD (1, 2),

Alette A. Koopman, MSc (1), Patrick van Schelven, MSc (1), Jefta van Dijk, MD (1), Johannes G.M. Burgerhof, MSc (3), Robert G.T. Blokpoel, MD (1)

(1) Department of Paediatrics, Division of Paediatric Intensive Care, Beatrix Children's Hospital, University Medical Center Groningen, The University of Groningen, Groningen, The Netherlands; (2) Pediatric Intensive Care Unit, University Hospital Heraklion, University of Crete, Greece; (3) Department of Epidemiology, University Medical Center Groningen, The University of Groningen, Groningen, the Netherlands; (4) Critical Care, Anesthesia, Peri-operative medicine & Emergency Medicine (CAPE), the University of Groningen, Groningen, the Netherlands

**Methods**

Set-up bench model

A Michigan Test Lung model 1601, composed of infant and adult lung chambers (Michigan Instruments, Grand Rapids, USA), was connected to an AVEA ventilator (Vyaire, Yorba Linda, CA, USA) with a non-heated, non–humidified breathing circuit and a cuffed ETT (Figure 1). ETT sizes with internal diameter (ID) 3.0mm to 8.0mm were used to match patient sizes of 5, 10, 20, 30, 50 and 70 kg body weight respectively (Table 1). Two types of conventional breathing circuits were used, a neonatal with diameter of 10mm (Intersurgical LTD, Berkshire, UK) connected to ETT sizes 3.0 and 4.0 mm (Halyard Health, Alpharetta, USA) and an adult with diameter of 22mm (Intersurgical LTD, Berkshire, UK) connected to ETT sizes 5.0 to 8.0 mm (Medtronic Covidien, Minneapolis, USA). This reflected clinical practice.

To simulate a wide range of physiologic and pathologic respiratory mechanic conditions, five levels of Crs were set, being 1 (normal), 0.8 (low), 0.6 (moderately low) 0.3 (severely low), and 0.15 ml/cmH_2_0/kg (extremely low). The ETT size 3.0mm extremely low Crs (<1ml/cmH_2_O) scenario was not performed, as Michigan Test Infant Lung was not adjustable at this level. Airway resistance (Raw) was simulated by restricting flow with linear pneumatic resistors (Series 7100, Linear type, Hans Rudolph Inc., USA) connected between ETT and lung simulator. Three levels of Raw were tested, being no, low and high resistance. Linear flow resistors resembling low and high Raw respectively were: for ETT 3.0mm and 4.0mm 50 and 200 cmH_2_0/L/s, for ETT 5.0 mm and 6.0 mm 20 and 50 cmH_2_0/L/s, for ETT 7.0 mm and 8.0 mm 5 and 20 cmH_2_0/L/s. Infant lung was ventilated under all aforementioned conditions with ETT sizes 3.0mm and 4.0mm, as well as in severely and extremely low Crs simulation with ETT 5.0mm and extremely low with ETT 6.0mm. The adult lung was used in all the remaining scenarios. Neonatal and adult flow sensors (Varflex, Vyaire, Yorba Linda, CA, USA) were positioned between the ETT and Υ-piece of the breathing circuit according to lung size.

Ventilator protocol bench study

PCV mode was used for all measurements. Each condition of Crs and Raw was tested setting three levels of pressure above PEEP (PAP) 10, 15 and 20 cmH_2_O and across three levels of inspiratory time (i.e., appropriate for age and ETT size, 20% below and 20% above this value) (Table 1). PEEP was kept constant at level of 5 cmH_2_O throughout the experiment. Respiratory rate was set to avoid intrinsic PEEP. Flow-time scalars were visually inspected for dynamic hyperinflation. Each measurement combination setting consisted of 15 consecutive breaths. All measurements were done by changing one variable at a time, while keeping all the others constant.

Data acquisition bench study

All data were acquired, synchronized and analyzed using a custom – build software program (Polybench, Applied Biosignals, Weener, Germany). A pressure transducer measuring the pressure at the Y-piece of the patient circuit and in the lung simulator was connected to a pulmonary function monitor (New Life Box, Applied Biosignals, Weener, Germany). Mechanical data were sampled at 200Hz and computed offline (Polybench, Applied Biosignals GmbH, Weener, Germany). Before recording experimental measurements, all pressure and flow sensors were zeroed to ambient pressure at sea level, calibrated and absence of leak was confirmed. Peak inspiratory pressure was measured at the Y-piece of the breathing circuit. Pplat was measured during a zero-flow state at the end of inspiration by an inspiratory hold maneuver for 4 seconds. Flow and Vt were measured using a proximally placed flow sensor (Varflex, Vyaire, Yorba Linda, CA, USA) that was connected to the AVEA ventilator, which in turn was connected via the analog output port to the computer using an analog to digital converter.

Measurements were made changing one variable at a time, keeping the others constant.

All data were acquired, synchronized, sampled at 200Hz and analyzed using a custom – build software program (Polybench, Applied Biosignals, Weener, Germany). Each scenario was recorded for up to 3 minutes with 15 consecutive breaths being analyzed. Mechanical energy delivered by the ventilator (MEventilator) was calculated by 0.098 * (Vt * kg^-1^) * (PIP – [(Pplat – PEEP)/2]). The mechanical energy delivered to the lung (MElung) was also calculated by integrating the area of the dynamic lung pressure – volume curve measured after the airway resistor. Baseline elastic energy was not included in the calculations because PEEP remained constant (5 cmH_2_O).

Statistical analysis

Multivariate linear regression analysis was used to identify independent contributors to the percentage difference between MEventilator and MElung. For these analyses, continuous variables including ETT size, compliance, resistance and inspiratory time were categorized. All statistical analyses were performed using software IBM SPSS, v24.0 (IBM Corp., Chicago, III, USA) with *P* < 0.05 considered statistically significant.

**References**

**Table 1**

| ETT size (mm) | 3 | 4 | 5 | 6 | 7 | 8 |
| --- | --- | --- | --- | --- | --- | --- |
| Weight (kg) | 5 | 10 | 20 | 30 | 50 | 70 |
| Ventilation mode |  | | | | | |
| Pressure controlled | PIP 10 – 15 – 20 cmH_2_O | | | | | |
| Volume controlled | Vt 5 – 7.5 – 10 mL/kg | | | | | |
|  | Inspiratory flow 0.5 – 1.0 L/kg/min | | | | | |
| Settings irrespective of ventilator mode | | | | | | |
| Breath rate (/min) | 15 | | | | | |
| Compliance (mL/cmH_2_O/kg) | 0.15 – 0.30 – 0.45 – 0.60 – 0.75 – 1.0 | | | | | |
| Resistance | None – moderate - high | | | | | |
| Inspiratory time ± 20% (sec) | 0.55 | 0.65 | 0.80 | 0.80 | 1.0 | 1.0 |

ETT endotracheal tube size; PIP peak inspiratory pressure; Vt tidal volume

**ESM Figure 1**

**
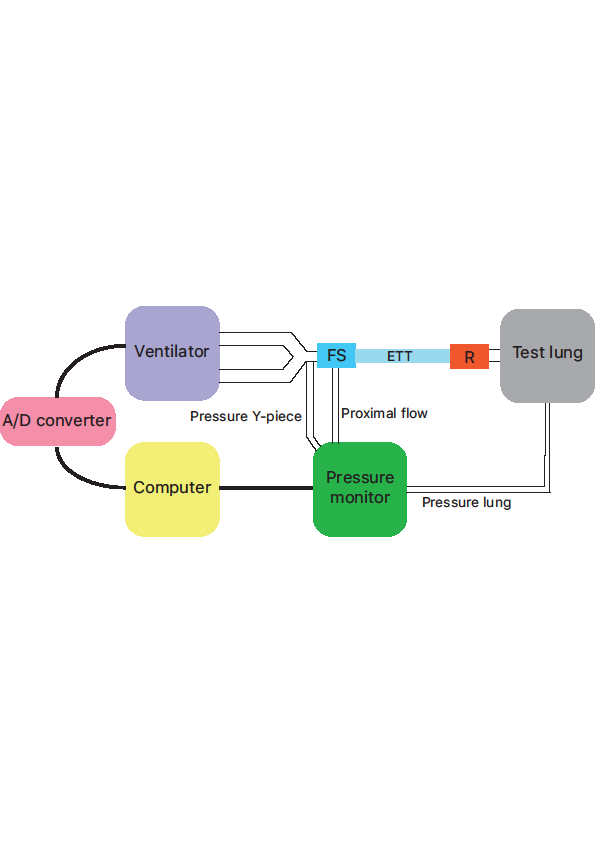
**

Schematic overview of the ventilation experimental set-up. Artificial infant and adult test lung (Michigan Test Lung, model 1601, Michigan Instruments, Grand Rapids, USA) was connected to the ventilator (CareFusion AVEA ventilator, Yorba Linda, USA) via the endotracheal tube (ΕΤΤ), the Y piece and the circuit. Resistance (R) was simulated by restricting flow with linear pneumatic resistors (Series 7100, Linear type, Hans Rudolph Inc., USA) connected between ETT and lung simulator. Flow sensors (FS), either infant or adult (Varflex, Vyaire, Yorba Linda, CA, USA), were inset after the Y-piece and connected to ventilator. Infant lung was ventilated using a 10 mm diameter breathing circuit system and adult lung was ventilated using a 22 mm (Intersurgical LTD, Berkshire, UK). Pressure transducers connected to an analog-to-digital converter (Bicore II, Applied Biosignals, Weener, Germany) transmitted data from Y-piece and intrapulmonary lung to the computer for future data analysis. Ventilator waveforms were stored through an analog output (A/D converter) to the computer.
